# Contralateral Total Hip Arthroplasty After Hindquarter Amputation

**DOI:** 10.1155/SRCM/2006/28141

**Published:** 2006-10-09

**Authors:** Scott M. M. Sommerville, James T. Patton, Jonathan C. Luscombe, Robert J. Grimer

**Affiliations:** The Royal Orthopaedic Hospital Oncology Service, Royal Orthopaedioc Hospital, Bristol Road South, Northfield, Birmingham B31 2AP, UK

## Abstract

We describe the management and outcome of a 62-year old lady who developed severe osteoarthritis of the hip, nine years after a hindquarter amputation for radiation-induced sarcoma of the contralateral pelvis. The difficulties of stabilising the pelvis intraoperatively and the problems of postoperative rehabilitation are outlined. The operation successfully relieved her pain and restored limited mobility.

## INTRODUCTION

We report our experience of performing a total hip arthroplasty for osteoarthritis of the hip in a patient who had previously undergone contralateral hindquarter amputation.

Patients with musculoskeletal sarcomas are now surviving their disease in greater numbers. They are clearly at risk of developing degenerative conditions like the normal population. In certain circumstances they may be at increased risk of developing degenerative arthritis due to the abnormal loads and motion occurring in joints adjacent to or left behind after major oncological surgery. There is some evidence to suggest that amputees have a higher incidence of osteoarthritis in remaining joints in both ipsilateral and contralateral limbs [1–4]. Although there is no data about the prevalence of osteoarthritis of the opposite hip, following a hindquarter amputation, it would not be an unlikely condition to develop.

There are very few reports on total joint arthroplasty in amputees, and these generally concern below knee amputees [3–5]. Hip arthroplasty following contralateral hindquarter amputation raises some interesting preoperative planning, operative technique, and rehabilitation issues. We will discuss the procedure and the rationale behind our approach to the problem.

## CASE REPORT

**Figure 1:**
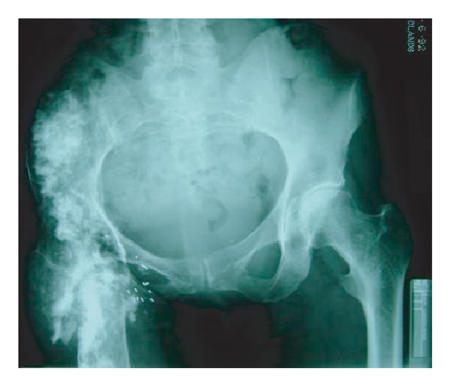
The radiological appearance of her right hip following previous TB, arthrodesis, subsequent lymphoma, and radiotherapy—and there is now a radiation induced sarcoma involving the right hemiplevis! Note that the left hip is normal.

In 1952, a female patient then aged 13 had a tuberculous infection of the right hip joint, which was subsequently arthrodesed. In 1974, then aged 36, she developed a non-Hodgkins lymphoma of the right femur which was treated with radiotherapy. In 1992, the patient, then 52 years old, was diagnosed with a high-grade spindle cell sarcoma involving the right proximal femur and extending into the right acetabulum (Figure 1). This was likely to have been a radiation-induced sarcoma. Due to the previous radiotherapy and the soft tissue extent of the tumour, a right hindquarter amputation was performed which achieved wide surgical margins. At the time of amputation there was no clinical or radiological evidence of left hip arthropathy, although there was evidence of a minor degree of acetabular dysplasia.

**Figure 2:**
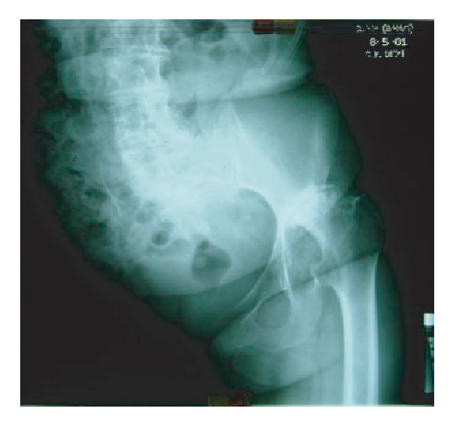
AP X-ray of the pelvis showing the result 9 years following hindquarter amputation, the marked degenerative changes of the spine, and the severe osteoarthritis changes of the left hip. It is apparent that the whole pelvis is tilted to the left.

Following amputation she remained well, mobile with crutches, and totally self-caring. A prosthesis was fitted but not used. Seven years following her amputation she started to complain of increasing discomfort in the left groin and thigh, with associated radiographic changes of osteoarthritis affecting the left hip. She also had low back pain. The arthritis in her hip progressed to a stage where she had become chair bound and had lost her social and functional independence. Nonoperative therapies had been exhausted and in 2001, when she was aged 62, a decision to perform a total hip arthroplasty was made (Figure 2).

Under a general and epidural anaesthetic, the patient was positioned on her right side with the spine parallel to the operating table and with the remaining hemipelvis vertical to this. The position was maintained with the aid of a suction beanbag. We attempted to maintain this lateral position with the use of a vacuum beanbag moulded to the patient's shape. A meticulous closure of the abductors was performed and the wound was drained for 24 hours. A broad-spectrum antibiotic was given on induction and for 24 hours postoperatively. No abduction pillow could be used. Mobilization was commenced on the fifth postoperative day and included the use of the hydrotherapy pool. She was safely mobile and able to be discharged on day 20 postoperatively.

**Figure 3:**
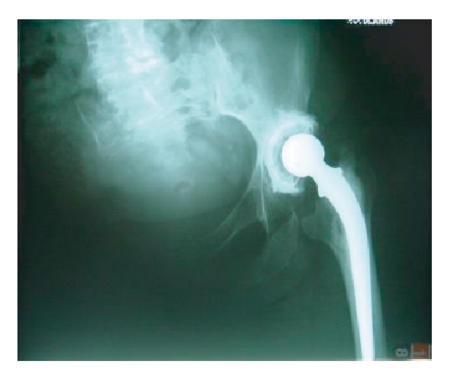
Three months following the left total hip arthroplasty. There appears to be a stress fracture through the floor of the acetabulum.

She was reviewed 3 months following surgery with sudden increase in pain around the hip. Radiographs revealed a fracture through the floor of the acetabulum (Figure 3). This was treated conservatively with rest in a wheelchair, although she was allowed to transfer independently.

**Figure 4:**
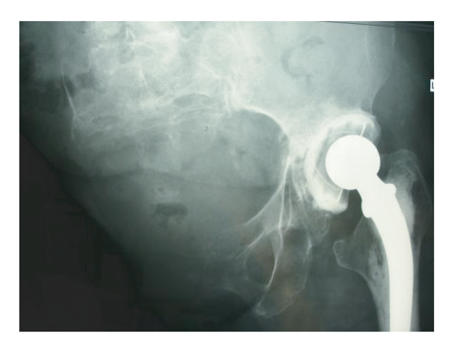
The radiological appearance of her pelvis 4 years after the total hip replacement. There has been some migration of the cemented cup but the hip causes her no pain.

When reviewed in 2005, four years after the hip replacement and now aged 66 she remained completely independent, living alone. She did her own housework and simple gardening (often sitting on the ground to do the weeding) and could do her own shopping. She could drive herself but her walking tolerance was only 50 meters and was limited by back pain. The replaced hip caused her no pain and examination showed it to have a full range of movements. X-rays showed that the fracture of the floor of the acetabulum had healed but the cup had migrated slightly proximally. She tended to have an adducted leg but there were no radiological or clinical features suggestive of subluxation (Figure 4).

## DISCUSSION

The treatment options for this patient were either nonoperative or operative. Her pain was only partially relieved by maximal doses of simple analgesics. Her mobility was deteriorating and her independence was at risk. It was felt that nonoperative treatment options had been exhausted. The only surgical options available were either hip arthrodesis or hip arthroplasty. Arthrodesis of the joint was dismissed due to her age, lumbar spinal arthritis, and the prolonged immobilization that would have been required. She would not have been able to mobilize until bony union had been achieved which may have taken several months.

Arthroplasty was considered the most appropriate surgical option. We could find no previous reported cases in the literature to offer guidance. A number of issues were identified and considered preoperatively. Firstly the positioning of the patient for surgery was considered. In total hip arthroplasty it is crucial to know the position of the pelvis so as to orient the cup appropriately. There was a strong argument for having the patient supine on the operating table, however we chose to place her on her side on a beanbag. Great care was taken to ensure the position of the remaining hemipelvis was as it would have been had she had both hemipelves. We felt that our familiarity with hip arthroplasty in this position would enable an easier and more rapid operation.

The operative approach was also a difficult decision. It was felt that this patient would be prone to dislocation due to the likelihood of repeated abnormal positioning of the hip joint. For example she would require significant flexion when arising from a chair, and adduction and flexion are likely if she lies on her right side. The remaining hemipelvis would also assume a position of abduction when weight bearing creating a relatively open acetabulum. For this reason we avoided the posterior approach and chose to approach the hip joint via a direct lateral approach. We accept that this approach endangers the abductor muscle function.

Although we took great care in positioning the patient, we felt that the exact orientation of the remaining hemipelvis may not have been absolutely accurate. The remaining hemipelvis may have fallen into a slightly adducted position. This combined with the slight acetabular dysplasia and significant superior acetabular erosion has resulted in an acetabular component that is in a more open position than was planned for preoperatively. Preoperatively the plan was to place the acetabular component more closed than one would normally choose in an attempt to minimize the chance of dislocation due to the abducted hemipelvis. Although dislocation has not presented a problem to date, the orientation of the acetabular component did cause us concern. In future we would consider positioning the patient supine on the operating table or using intraoperative radiography to assess pelvic positioning. A 32 mm head size was chosen also in an attempt to reduce the likelihood of dislocation, although we accept that the relationship of head size and dislocation is unproven [6].

The patient was mobilized on the fifth postoperative day, which is quite delayed from our usual rehabilitation protocol. She was mobilized with care and the hydrotherapy pool played an integral role in her rehabilitation. It minimized the load on the abductors and allowed these muscles to gradually increase in strength whilst still protecting the repair and allowing mobilization. She was able to be discharged by day 20 postoperatively. Protracted hospital stays have been reported previously in amputees undergoing total joint arthroplasty [5].

We are uncertain whether this total hip arthroplasty will have the similar excellent survival to that reported previously for the Stanmore total hip prosthesis [7, 8]. It may be that her abnormal gait results in abnormal forces through this joint leading to premature aseptic loosening. The literature on total joint arthroplasty in amputees is very limited. Prickett and Scanlon [3] discussed an ipsilateral total hip arthroplasty and a total knee arthroplasty performed in below knee amputees. The total hip arthroplasty was performed for a fracture and the total knee arthroplasty for osteoarthritis. The report does not discuss the operative details at all, but rather focuses on the postoperative rehabilitation. Pasquina and Dahl [5] detail the rehabilitation following ipsilateral total knee arthroplasty in a below knee amputee. The total knee arthroplasty was performed for osteoarthritis. One year later the patient underwent contralateral total knee arthroplasty. There is also some discussion on the preoperative considerations and planning of such a procedure. The end result appeared to be excellent and they concluded that individuals with amputations should be considered for total joint arthroplasty. Subsequently Salai et al [4] presented a series of five amputees who underwent total hip arthroplasty via a direct lateral approach. All were performed following a fractured neck of femur with three being performed after failed internal fixation and two as the primary treatment. All had ipsilateral below knee amputations, although one patient was a bilateral amputee with a contralateral above knee amputation as well as the below knee amputation. This patient had a good result following arthroplasty being able to ambulate with a single cane and two prostheses postoperatively. They recommend the procedure for any amputee with a fractured neck of femur, as they believe the results of internal fixation in these patients is poor and the results of arthroplasty in this small series of patients are good.

The development of the fracture through the floor of the acetabulum was unexpected and difficult to deal with. We suspect it is an insufficiency fracture due to the relative osteoporosis of the lower part of the pelvis on the remaining side. The increased stresses put upon this by the cemented hip replacement probably led to the fracture. This could potentially have been a problem with any type of acetabular component. It would probably also have arisen in an uncemented cup and may in fact have led to loosening of this at a very early stage. The fact that the pelvic ring is “open” following a hindquarter amputation probably produces a relative osteoporosis of the lower half of the remaining hemipelvis which may well have accounted for the fracture.

## CONCLUSION

Total joint arthroplasty in amputee patients is a rarely performed procedure, but is one that is likely to become more frequent. We have detailed what we believe is the first reported case of a total hip arthroplasty in a hindquarter amputee. We have discussed our preoperative planning, surgical technique, and postoperative rehabilitation. Suggestions for improvements for our technique have been offered. Whilst total joint arthroplasty in amputees is currently unproven, we believe it to be of great benefit to those with concurrent degenerative joint disease. Early results are sufficiently encouraging enough for us to recommend this procedure.
